# Strategies for deploying triple artemisinin-based combination therapy in the Greater Mekong Subregion

**DOI:** 10.1186/s12936-023-04666-4

**Published:** 2023-09-06

**Authors:** Freek de Haan, Chanaki Amaratunga, Van Anh Cao Thi, Long Heng Orng, Manithong Vonglokham, Thieu Nguyen Quang, Dysoley Lek, Wouter P. C. Boon, Arjen M. Dondorp, Ellen H. M. Moors

**Affiliations:** 1 Copernicus Institute of Sustainable Development, Utrecht University, Utrecht, The Netherlands; 2 Mahidol Oxford Tropical Medicine Research Unit, Faculty of Tropical Medicine, Mahidol University, Bangkok, Thailand; 3 Center for Tropical Medicine and Global Health, Nuffield Department of Medicine, University of Oxford, Oxford, UK; 4 The University of North Carolina Project in Vietnam, Institute for Global Health and Infectious Diseases, University of North Carolina at Chapel Hill, Chapel Hill, USA; 5 Mahidol Oxford Tropical Medicine Research Unit, Epidemiology Department, Mahidol University, Bangkok, Thailand; 6 Lao Tropical and Public Health Institute, Ministry of Health, Vientiane Capital, Lao PDR; 7 National Institute of Malariology, Parasitology and Entomology, Hanoi, Vietnam; 8 National Center for Parasitology, Entomology and Malaria Control, Phnom Penh, Cambodia

**Keywords:** Triple artemisinin-based combination therapy (TACT), Implementation strategies, Antimalarial resistance

## Abstract

**Background:**

This is a qualitative study to identify implementation challenges for deploying triple artemisinin-based combination therapy (TACT) in the Greater Mekong Subregion (GMS) of Southeast Asia and to explore strategies to overcome these challenges.

**Methods:**

In-depth interviews were conducted in three countries that have repeatedly been confronted with ACT failures: Cambodia, Vietnam, and Lao PDR. Thirty-nine key stakeholders in the healthcare systems in these countries were interviewed. One participatory workshop was conducted in Cambodia, where scenarios for potential TACT deployment were discussed.

**Results:**

The results section is organized around four strategic themes that emerged from the data: policy support, data and evidence, logistics and operation, and downstream engagement. The study revealed that countries in the GMS currently rely on ACT to eliminate *Plasmodium falciparum* malaria by 2025. TACT is, however, considered to be a useful backup strategy in case of future treatment failures and to prevent the re-establishment of malaria. The study showed that a major challenge ahead is to engage decision makers and healthcare providers into deploying TACT, given the low case incidence of falciparum malaria in the GMS. Interview respondents were also skeptical whether healthcare providers would be willing to engage in new therapies for a disease they hardly encounter anymore. Hence, elaborate information dissemination strategies were considered appropriate and these strategies should especially target village malaria workers. Respondents proposed several regulatory and programmatic strategies to anticipate the formation of TACT markets in the GMS. These strategies include early dossier submission to streamline regulatory procedures, early stakeholder engagement strategies to shorten implementation timelines, and inclusion of TACT as second-line therapy to accelerate their introduction in case they are urgently needed.

**Conclusions:**

This paper presents a qualitative study to identify implementation challenges for deploying TACT in the GMS and to explore strategies to overcome these challenges. The findings could benefit researchers and decision makers in strategizing towards potential future deployment of TACT in the GMS to combat artemisinin and partner drug resistance.

## Background

The prevalence of *Plasmodium falciparum* malaria in Southeast Asia is at historically low levels and the region is collectively engaging in malaria elimination strategies [1]. Concurrently, the Greater Mekong Subregion (GMS) is meeting the challenge of resistance to artemisinin and partner drugs used in artemisinin-based combination therapy (ACT). Artemisinin-based combinations are the global first-line therapies for the treatment of uncomplicated malaria and losing them to resistance would jeopardize malaria control and elimination activities worldwide [2].

Artemisinin and partner drug resistance and subsequent ACT failures force countries in the GMS to anticipate new treatment strategies to protect their malaria control and elimination ambitions [3]. Countries rotate artemisinin-based combinations, when treatment failures are observed, but rotation has repeatedly proven a temporary solution before the replacement ACT also fails [4]. Other proposed strategies include the simultaneous deployment of multiple first-line therapies, or extending ACT use from 3 to 7 days [5, 6]. However, these solutions have been associated with significant operational challenges and limited feasibility.

Another option that is now being explored in the GMS and elsewhere is the introduction of *triple* artemisinin-based combination therapy (TACT) [7, 8]. The rationale is that combining the artemisinin derivative with two carefully selected partner drugs will extend the therapeutic lifetime of each compound because the parasite will need to develop resistance to three drugs instead of two. The potential benefits of introducing TACT would be twofold: 1) they can provide direct clinical relief in case all current artemisinin-based combinations (including newly introduced artesunate-pyronaridine) would fail, and 2) they can protect artemisinin and its partner drugs from resistance, preserving future treatment options.

Although results from current clinical trials [7, 9] and mathematical modelling studies [10, 11] are encouraging, the introduction of TACT in the GMS is debated [12, 13]. Some scholars and representatives of policy institutes perceive TACT as a useful intervention towards malaria elimination in the GMS, while others prefer relying on current strategies of rotating ACT until malaria elimination is established. A recently conducted Delphi study systematically assessed expert perspectives towards the introduction of TACT in Southeast Asia [14]. Prominent malaria experts identified major advantages, disadvantages and implementation barriers for introducing TACT and they rated the relevance of each item on a 5-point Likert scale. The insights of the Delphi study led to a first, tentative overview of major barriers and drivers towards TACT deployment in the GMS. However, these insights lack contextualization to the individual countries. More in-depth attention can—and should—be paid to the specific implementation challenges for deploying TACT in the GMS and to strategies to overcome these challenges [15]. This study addresses this gap in literature through a qualitative study of implementation challenges and deployment strategies of TACT in three countries in the GMS: Cambodia, Vietnam and Lao PDR.

## Methods

### Research design

The study was conducted under the auspices of the UK Government’s Foreign, Commonwealth & Development Office funded Development of Triple Artemisinin Combination Therapies (DeTACT) project. A qualitative research approach was employed to investigate implementation challenges and deployment strategies for TACT in the Greater Mekong Subregion (GMS) of Southeast Asia. Three countries were selected for data collection: Cambodia, Vietnam, and Lao PDR. All three countries are engaging in malaria elimination strategies, have repeatedly been confronted with artemisinin and partner drug resistance, yet they all have unique health system characteristics. In-depth interviews were conducted in all three countries to obtain insight into specific implementation challenges for TACT and to explore strategies to overcome these implementation challenges. The multi-country research approach enabled investigating country-specific dynamics in relation to the deployment of TACT, while enabling the extraction of general topics that were applicable to more than one country. Data was collected through in-depth interviews with key actors in the healthcare systems in Cambodia, Vietnam and Lao PDR. The implementation challenges for introducing TACT in Southeast Asia identified in the previously conducted Delphi study [14] was considered as a starting point for the in-depth interviews (Table 1). Furthermore, one participatory workshop was conducted in Cambodia. The goal of the workshop was to interactively discuss preliminary insights obtained during the interviews with key stakeholders and to discuss strategic solutions towards the deployment of TACT in the GMS.

**Table 1:** Expert perspectives on the *implementation barriers* for introducing TACTs in Southeast Asia (derived from de Haan et al. [14])

Implementation barrier	Explanation
Intensified prescriber training	Intensifying training requirements for correct TACT prescription
Donor funder support	Obtaining support by donor funders to cover TACT implementation costs and potential price increases
National policy support	Obtaining support from national malaria control programs and other national decision makers to engage in the deployment of TACT
WHO and global policy support	Obtaining support from the WHO and other global decision makers to engage in the deployment of TACT
Availability of fixed-dose combination (FDC) TACT	Ensuring timely development and production of fixed-dose combination (FDC) for TACT
Community acceptance	Ensuring community acceptance by providing clear communication and tackling potential misconceptions about TACT
Collecting safety and efficacy data	Collecting sufficient efficacy and safety data to support the introduction of TACT
Supply chain logistics	Adapting import, procurement and supply routes for the introduction of TACT
Regulatory approval	Obtaining timely regulatory approval for introducing TACT in Southeast Asia
Set up surveillance systems	Setting up surveillance systems to monitor drug resistance rates and adherence to TACT
Private sector engagement	Engaging the (informal) private sector in TACT deployment and creating demand beyond official programs
Set up pharmacovigilance systems	Setting up a pharmacovigilance system for TACT
Stockpile management	Managing stockpiles for countries that still have ACT stocks or contract deals with ACT producers

### Respondent selection

The study was conducted in Cambodia, Vietnam and Lao PDR. In each of the countries, collaborations were established with co-authoring research institutes and social scientists. The local social scientists mapped potential respondents and invited them to participate in the in-depth interviews. The aim was to include interviewees who represented a wide variety of stakeholders in anti-malarial drug transitions. Selected respondents included representatives from national malaria control programmes, regulatory authorities, academia, healthcare professionals and NGOs. Sampling of respondents in each country continued until reaching data saturation on the pre-identified implementation barriers for the deployment of TACT.

### Data collection

Preparatory meetings were held between the principal investigators (FH and CA) and the social scientists in Cambodia (LO), Vietnam (VC) and Lao PDR (MV). The purpose of these meetings was to discuss the research aims, identify relevant stakeholders and prepare data collection tools. Semi-structured interview guidelines were designed using the pre-identified implementation barriers as starting questions. In line with insights gained in the ongoing DeTACT project, the study focused on the introduction of a prospective TACT that combines artemether-lumefantrine plus amodiaquine (AL + AQ). For each interview, themes that were considered most relevant to the specific background of the respondent were selected and included in a personalized interview guideline. Pilot interviews were conducted to improve mutual understanding and to reduce ambiguity. Using the semi-structured interview guides enabled exploring the same topics between the countries and respondents, while remaining flexible for newly emerging themes. Data collection was iterative: insights from previous interviews were incorporated in guidelines of later interviews.

### Data analysis

Interviews were recorded with consent given by each respondent. All interviews in Cambodia, Vietnam and Lao PDR and the participatory workshop in Cambodia were transcribed verbatim and translated into English by the social scientists or by professional translators. Each transcript was then uploaded to NVivo 12 software and subjected to coding. The transcripts were coded line-by-line by FH, and codes were assigned to both the pre-defined implementation barriers and to newly emerging themes. After the process of coding, a thematic analysis was employed using deductive and inductive techniques: emerging themes were merged into overarching categories and storylines were written to present narratives of implementation challenges and deployment strategies to overcome these implementation challenges.

### Study setting: introducing the three country contexts

Cambodia, Vietnam and Lao PDR have low falciparum malaria incidence and all three countries are engaging in malaria elimination strategies. In 2021, Cambodia (16.6 million inhabitants) reported 4.382 malaria cases, Lao PDR (7.4 million inhabitants) reported 3.897 malaria cases and Vietnam (97.5 million inhabitants) reported 377 malaria cases (WHO, 2022). These numbers include both *Plasmodium falciparum* and other types of malaria. All three countries share goals to eliminate falciparum malaria by 2025 and all other types of malaria by 2030 [16].

Cambodia: Cambodia has repeatedly changed its first-line ACT as a response to failures for the treatment of uncomplicated falciparum malaria. In 2017, artesunate-mefloquine (ASMQ) was re-introduced as first-line therapy in response to treatment failures with dihydroartemisinin-piperaquine (DHA-PPQ). At the time of writing, ASMQ maintained adequate treatment efficacy in Cambodia, but the national malaria control programme is preparing for the implementation of artesunate-pyronaridine (AS-PYR) as first-line therapy. The National Center for Parasitology, Entomology and Malaria Control (CNM) coordinates malaria-related activities and the Department of Drugs and Food (DDF) is responsible for drug regulation in Cambodia.

Vietnam: The official first-line therapy for the treatment of uncomplicated falciparum malaria in Vietnam is DHA-PPQ. However, DHA-PPQ is failing in some areas in the central-highlands and AS-PYR is being used for the treatment of uncomplicated falciparum malaria in these areas. From November 2022 onwards, AS-PYR was used throughout the entire country. Malaria control activities in Vietnam are coordinated by the National Malaria Program (NMP) and the Drug Administration of Vietnam (DAV) is responsible for drug regulation in Vietnam.

Lao PDR: The first-line therapy for the treatment of uncomplicated falciparum malaria in Lao PDR is artemether-lumefantrine (AL). Moreover, AS-PYR and ASMQ were added to national treatment guidelines as second-line therapy in 2022. In Lao PDR, malaria control efforts are coordinated by the Center of Malariology, Parasitology and Entomology (CMPE) and the Food and Drugs Department (FDD) is responsible for drug regulation.

## Results

A total of 39 interviews were conducted (12 in Cambodia, 12 in Vietnam and 15 in Lao PDR) between May and December 2022. Selected respondents included representatives from national malaria control programmes, regulatory authorities, academia, healthcare professionals and NGOs. The goal of the interviews was to explore pre-defined implementation challenges (based on Table 1) and deployment strategies for TACT in the selected countries. Furthermore, a participatory workshop with 11 participants from Cambodia was held in Phnom Penh in October 2022. The goal of the workshop was to interactively discuss preliminary insights obtained during the interviews, to validate emerging insights, and to further explore strategic solutions towards implementation challenges for TACT. This results section is organized around four strategic themes regarding TACT introduction that emerged from the data: (1) policy support, (2) data and evidence, (3) logistics and operation, and (4) downstream engagement.

### Policy support

A positive attitude towards the deployment of TACT was expressed by almost all respondents in Cambodia, Vietnam and Lao PDR. TACT were considered a promising backup strategy in case current first-line ACT would start to fail. At the same time, some respondents expressed concerns related to exposing the parasite to three drugs, which may jeopardize future efficacy of these compounds.

Introducing TACT is a way to address drug resistant malaria. […]. If we use TACTs, the chance of genetic change and resistance to the three compounds will be reduced and we will have higher efficacy of treatment. However, we do not have many alternatives for malaria treatment, therefore we need to be careful in using the existing malaria drugs. (Lao PDR, #3)

It was emphasized that current first-line artemisinin-based combinations are still effective in all three countries. Most respondents expected to rely on their current artemisinin-based combinations, including newly introduced AS-PYR, for reaching the regional ambitions to eliminate falciparum malaria by 2025. Hence, the introduction of TACT was considered not a direct, urgent need, but rather a useful strategy in case alternative treatments would fail. In contrast to the other two countries, Cambodia is actively anticipating the introduction of TACT. During the workshop it was mentioned that decision-makers in Cambodia are considering AS-PYR as first-line therapy, after which TACT could become second-line therapy once available and pre-qualified by the World Health Organization (WHO). This would enable Cambodia to accelerate their introduction if treatment failures would occur with AS-PYR.

So in the future it is necessary to have a drug so that when Plasmodium falciparum becomes resistant to Pyramax [brandname of artesunate-pyronaridine], we still have an effective and safe drug to treat people who have malaria. (Vietnam, #1)

In Cambodia, the CNM [National Center for Parasitology, Entomology and Malaria Control] has decided to include TACTs in the malaria elimination policy already. The WHO also agreed that Cambodia can include TACT as second line therapy. The CNM has to use all resources to reach the elimination goals. (Cambodia, #1)

Some respondents in Cambodia, Vietnam and Lao PDR indicated that implementing new malaria therapies can be a lengthy process. Expected timelines of several months to more than a year were proposed from inclusion in national treatment guidelines to full implementation on the ground. This was considered potentially too long when dealing with problems of drug resistance. References were made to the slow implementation of other malaria interventions, in particular to the introduction of tafenoquine for *Plasmodium vivax* malaria in Vietnam, and the switch from DHA-PPQ to ASMQ in Cambodia. It was suggested that lessons should be learned from these past experiences: the introduction of TACT should be initiated well in advance and in accordance with key stakeholders such as regulators, national decision-makers and healthcare providers.

There is need for many documents to support the decision makers. I think it will take more than a year. (Lao PDR, #2)

If we want to change from ACT to TACT, it will take a lot of time, it can be 2 years, from revising guidelines to full implementation. (Cambodia, workshop)

Some respondents were skeptical of whether engaging in TACTs would be worth the investment in this pre-elimination era. Malaria incidence is receding and nearly all respondents expected that falciparum malaria in the GMS will be eliminated in the next few years. At the same time, it was mentioned that even if falciparum malaria is eliminated in 2025, effective therapies still need to be maintained in stock in case a resurge of malaria incidence occurs. Respondents did foresee a role for TACTs as such a back-up therapy.

Therefore, the market is very small, and getting smaller as the number of infected cases declines. So, introducing a new drug, or even conducting the trial, is difficult. (Vietnam, #10)

[…] Like in China: they may encounter malaria drug resistance imported from Southeast Asia. So China needs to have drugs in stock and needs to be alert. (Cambodia, #5)

Respondents in Cambodia, Vietnam and Lao PDR agreed that support from international institutions, in particular the WHO, would add to the credibility of introducing TACT. National decision-makers said they are unlikely to recommend a new therapy that is not endorsed by the WHO. The WHO is considered as the guiding institute and their recommendations provide guidance for country-level strategies against resistance. Nevertheless, it was indicated that national governments could deviate from general recommendations in case of an emergency situation. Interview respondents furthermore emphasized that procurement subsidies from the Global Fund to fight Aids, Tuberculosis and Malaria (GFATM) would add to TACT’ credibility and would be important for their affordability nationwide in case they would become deployed as first-line therapy.

I think as a general rule, health authorities are always more keen to do something if it's backed up by WHO recommendations. (Cambodia, #11)

In terms of malaria, then the role of the sponsor is vital, I mean the Global Fund. As you know, about 80% of the funding for malaria prevention, control and elimination comes from the Global Fund. (Vietnam, #2)

### Data and evidence

Safety and efficacy data are needed to support the market authorization and subsequent introduction of TACT in Southeast Asia. Respondents in Cambodia and Vietnam expressed a preference of such data collected in their own countries, because this would add to the credibility of the therapy within local contexts. However, they also acknowledged difficulties in collecting clinical evidence in the context of the receding malaria incidence. Some interviewees in Cambodia and Vietnam referred to the previous TACT-CV study in which good efficacy and safety of the TACT artemether-lumefantrine plus amodiaquine was demonstrated in Cambodia and Vietnam [7]. They suggested this evidence as a starting point for obtaining market authorization for TACT.

I assume they would ask for safety data, obtained from participants from Southeast Asia, and perhaps even from Cambodia. (Cambodia, #11)

With such a small number of cases, it will be hard to evaluate treatment effects. Previously in the study from 2015 to 2018, there were 4000 to 5000 cases in Vietnam and the majority was plasmodium falciparum. […]. But now, it will be more difficult to have a sample size of a few hundred to evaluate the effectiveness and safety [of TACTs]. (Vietnam, #10)

Cambodia, Vietnam and Lao PDR all operate under the Association of Southeast Asian Nations (ASEAN) regulatory system. Stringent regulatory approval such as WHO pre-qualification was considered an important driver for registration in the countries. Respondents in Vietnam indicated that AS-PYR is currently being deployed through a special import licence, which was obtained to enable its use prior to registration. This was deemed necessary when high levels of treatment failures with DHA-PPQ were observed. Given the urgency, there was no time to go through extended regulatory procedures and AS-PYR was immediately needed to treat patients with drug-resistant infections. It was suggested that a similar procedure could be followed for the introduction of TACT in case they are urgently needed in Vietnam. In Cambodia and Lao PDR no references to this type of regulatory shortcuts were made.

At the moment, Pyramax [brand name of artesunate-pyronaridine] is used in Vietnam as an unregistered drug. It is not registered to be distributed in Vietnam, but it is being imported according to a special licence. It does not have the market authorization, so it is called off-label medicine. (Vietnam, #10)

Timelines for obtaining regulatory approval and market licences varied between countries. Respondents in Vietnam and Lao PDR expected that they would need several months up to several years for regulatory procedures, going from dossier submission to obtaining prescription licences. Shorter timelines of approximately 1 month were expected for the regulatory trajectory in Cambodia, as long as complete dossiers would be submitted. One respondent from Vietnam indicated that as long as TACT are deployed as single tablets, their registration is rather straightforward and less time consuming. This is because all the individual compounds of the TACT are already registered. Registration of a potential fixed-dose combination (FDC) TACT would require more extensive regulatory procedures and timelines.

The process of approving and regulations for TACTs will take about 6-12 months, dependent on the availability of the documents. (Lao PDR, #1)

Respondents in Cambodia and Lao PDR expressed concerns about the administrative burden of a prospective transition to TACT. National treatment guidelines need to be adjusted, pharmacovigilance systems need to be setup and malaria surveillance procedures need to be adjusted. Furthermore, data obtained by pharmacovigilance and surveillance systems need to be collected and adequately processed. Respondents indicated budget restraints and a lack of human resources, which would complicate these procedures. Challenges related to broader information management practices and IT systems were also mentioned as possible challenges to adequately follow-up on a change in treatment practices. One respondent in Cambodia opposed this view, and was rather confident that routines could easily be adjusted, referring to the surveillance system of Cambodia as the most elaborate and most experienced in the world.

We will provide the training to staff to work on the pharmacovigilance systems and work with DDF about side effects of drugs. I know it is difficult to train and report on those cases, it takes time. (Cambodia #1)

So I forgot to tell you something about the surveillance formation system which we have in Cambodia I think that is one of the best in the region, I will not be lying to you if I say it is the best one in the world. (Cambodia #2)

### Logistics and operations

Interview respondents in Cambodia, Vietnam and Lao PDR were generally optimistic about the operational aspects of introducing TACT. Some logistical and operational challenges were nevertheless mentioned. Several respondents again referred to the low malaria incidence in this pre-elimination era in which key stakeholders stated that malaria is not considered a major health issue anymore. They pointed out challenges in engaging suppliers and local health staff and also challenges in mobilizing resources for procuring and distributing TACT. Other respondents expressed concerns about high implementation costs for introducing a new therapy and questioned if resources should not be invested in elimination activities instead.

There are costs that we have to think about. When you are introducing a new drug into a country, then you have to change your treatment guidelines, train human resource, fill the supply chain […], so we need more resources, and not only for procurement and distribution of the drug. (Cambodia, #2)

Every time that we update or change policy the first challenge is funding, we will need a huge budget for improving the policy and training healthcare providers in the country. (Lao #7)

General challenges in malaria management in Southeast Asia were mentioned as potential challenges for delivering TACT to patients. Malaria in Southeast Asia is mostly common among hard-to-reach populations such as migrant populations and illegal labor forces. These people often operate beyond the scope of the official channels and reaching them with appropriate interventions is considered complicated. This was brought forward in each country as an existing challenge for malaria case management that would also apply to TACT. Furthermore, respondents emphasized that stockpile issues should be considered before implementing TACT, including storage conditions, temperature requirements, climate control and expiry dates. These stockpile issues could pose challenges especially in light of relatively small quantities needed for TACTs in the GMS.

A challenge is the limitation of quantity and quality of the human resources. Ownership and knowledge of the staff at all levels is required but this is also a challenge. In particularly for supply, distribution and reporting. (Lao PDR, #1)

Now there are a few things that may be difficult in monitoring, detection, prevention, and treatment. The first thing is that, as I mentioned from the beginning, the at-risk people are in remote areas and mobile. They are migrant population from the North to across the border without declaration to the local authorities, which is difficult for early detection and timely treatment and is a risk of an outbreak. The second thing is that now the malaria is no longer endemic in large areas. (Vietnam, #7)

Another challenge is the coordination of many stakeholders: donors, purchasers and factories. Document processing is time consuming, and so sometimes the drugs nearly reach their expired dates when arrive in our country, and sometimes it is already beyond the expired date after distributed to provinces or districts. (Lao PDR, #2).

Several respondents doubted whether pharmaceutical companies would be prepared to engage in TACT if they are targeted exclusively to the GMS. They questioned whether TACTs would be considered profitable given the low malaria incidence in the GMS. Parallels were drawn with recent challenges encountered in Cambodia with the procurement of pediatric doses of ASMQ. When Cambodia switched to ASMQ in 2017, they had to order stocks at a private sector company who intended to deliver ASMQ at a minimum amount of half a million doses. However, only a fraction of this number was needed given the low malaria prevalence in the country, which resulted in wastage of unused drugs. During the participatory workshop in Cambodia, a regional procurement system for anti-malarials in the GMS was suggested as a potential solution to such stockpile issues in case multiple countries would collectively engage in TACT.

It is difficult because of the quantity of drugs. For example, for the current ACT we must use the foreign content, because they do not produce them domestically anymore due to the reduction of malaria. The requested quantity of drugs is not enough to balance the cost of production. So they stop producing the drug. (Vietnam, #5)

I just want to share experience during last time, Cambodia changed the treatment guideline after the piperaquine started to fail and we shifted to ASMQ. So at that time, we needed to buy from a factory by at least half million doses. If less than half million dose, the factory would not produce. […]. Now, maybe Cambodia is a first country to use this TACT. Simultaneously, malaria case in Cambodia sharply drops and there are few cases left. How to convince the manufacturer to produce TACTs? (Cambodia, workshop)

Respondents in all three countries indicated that public sector supply chains for anti-malarials are well-established due to many years of experience. Of the three countries, Lao PDR is the only country in which the private sector plays a major role in malaria management. In Vietnam and Cambodia, private sector clinics, hospitals and pharmacies are only involved in case detection, yet they are no longer permitted to prescribe anti-malarial therapies. Some respondents in Lao PDR emphasized that the private sector should be actively involved in the potential introduction of TACT to ensure their proper usage and to avoid misuse. References were made to the Public–Private Mix (PPM) programme as a promising instrument to engage private sector actors in Lao PDR. Through the PPM programme, private sector prescribers receive training and instructions by their public-sector counterparts, which enables them to act in accordance with treatment guidelines.

Currently, malaria drug treatment is free, and is supported by international organizations. I think it should not be sold in general [including private sector prescribers], because it will be difficult to follow up and it may lead to drug resistance. But we can discuss about the service fee of the private sector that participated in the PPM project. (Lao PDR, #5)

In my opinion, closely supervising them (private sector) regarding to diagnosis and reporting can help. Moreover, if we have incentives for them, it will be a factor in encouraging them to participate. (Lao PDR #10)

### Downstream engagement

Several respondents in Cambodia, Vietnam and Lao PDR indicated that prescriber training will always be required once a new therapy is introduced, and the introduction of TACT would be no exception. Such training programmes should include information about potential adverse effects, such as the increased risk of vomiting. It was also emphasized that the rationale for introducing TACT in terms of addressing drug resistance should be made explicit in training modules. Respondents in all three countries highlighted that training programs would especially need to target village malaria workers (VMWs), because they are at the frontline for malaria management in Cambodia, Vietnam and Lao PDR.

With the reduced number of cases we need to ensure that they get picked up early and they are given adequate treatment in a timely manner. And that is harder and harder where health professionals are not seeing malaria that much. And that is why malaria is not always on the top of their differential diagnosis. (Cambodia, #3)

The training needs to be detailed to the reason why we change to TACT. The training would have to focus on the 5 provinces in the southern part of Lao. (Lao PDR, #1)

Pricing issues were considered of limited importance for engaging community members in TACTs. All anti-malarial therapies in Cambodia and Vietnam, and most anti-malarial therapies in Lao PDR, are available for free through public sector channels. Hence, financial considerations generally play a minor role in the adoption decisions of community members. Respondents furthermore indicated that malaria patients generally comply to prescribed therapies when diagnosed with malaria. Side-effects and the number of pills to be taken per day were mentioned as factors that could negatively affect acceptance of TACT. Some respondents expected higher rates of adverse effects such as vomiting and nausea for a TACT of AL + AQ because of the inclusion of the amodiaquine compound. Thorough explanation of side-effects through information leaflets or prescriber explanations would be required according to them.

I think there is no problem about community acceptance, because the community does not know what this drug is, whether it is new or old, as long as it is prescribed, right? The doctor prescribes and the patient will take it. But the most important thing is that it's only for short term and must be fixed-dose in the one tablet. (Vietnam, #2)

The important thing is we explain to community; explain to community members or patients the side effects of the drug and the benefits of using the new drug. As for the side effects of the drug, how does it affect the patients and what are the benefits to the patients. (Cambodia #10)

Respondents in all three countries emphasized that fixed-dose combinations (FDCs) would be preferred over separate tablets. Prescribing FDCs would enhance patient compliance and would be more convenient for prescribers. Moreover, some respondents referred to other ACT regimens with separate tablets to explain risks of non-compliance to partner drugs that are associated with adverse effects. Most respondents stressed that the number of pills would be an important reason for preferring FDC TACT: more pills will increase risks of non-compliance. Some references to non-compliance of earlier single tablet ACT regimens were made by respondents from Cambodia and Lao PDR.

As a past experience has shown, control programs are much happier when the drug is in a single pill rather than having to separated or co-blistered or whatever. It's easier in terms of implementation in the field. And that would also be the case for TACTs. (Cambodia, #11)

I think, in fact, the fixed-dose combination will have more advantages because taking one dose including three ingredients in a pill will enhance drug adherence, avoid forgetting medication, and it will be more convenient for patients. (Vietnam, #11)

## Discussion and conclusions

Countries in Southeast Asia are experiencing a historically low malaria burden and they are increasingly dedicating resources and efforts towards the elimination of falciparum malaria [16]. Respondents in Cambodia, Vietnam and Lao PDR indicated a general reliance on their current ACT in reaching their malaria ambitions. In particular, newly introduced artesunate-pyronaridine (AS-PYR) was mentioned as an important intervention towards malaria elimination. Although malaria elimination is without doubt the most effective way to contain drug resistance, there is a risk of over-relying on current elimination strategies while alternative scenarios are being overlooked. Resistance pathways are unpredictable and epidemiological developments can unfold rapidly [3]. For example, the instable political situation in Myanmar and subsequent weakening of health services could result in a resurgence of falciparum malaria not only in Myanmar, but also in neighbouring countries in the GMS [1]. Moreover, future treatment failures with AS-PYR can potentially emerge and can threaten malaria elimination ambitions when countries remain unprepared. Therefore, decision makers would benefit from an increasing number of treatment options. Triple artemisinin-based combination therapy (TACT) is currently being developed and it has the potential to ensure treatment efficacy in case ACT would fail [7, 17], while they can also protect drug compounds from resistance, increasing future treatment options.

This qualitative study was conducted to identify implementation challenges for TACT deployment in the GMS and to explore strategies to overcome these implementation challenges. Data were collected in three GMS countries that have repeatedly been confronted with ACT failure: Cambodia, Vietnam and Lao PDR. The results were organized around four strategic themes.

The first strategic theme that emerged from the data was policy support for the deployment of TACT. This study revealed that early stakeholder engagement is essential to prevent implementation delays for new therapies such as TACT. These stakeholder engagement strategies should be encompassing and should target decision-makers, regulators and supply chain actors. Indeed, it is widely acknowledged that transitioning to new malaria therapies can be a lengthy process [18, 19] and that early stakeholder engagement is a driving force towards accelerated uptake of malaria interventions [20, 21]. In contrast to Vietnam and Lao PDR, Cambodia is already anticipating the potential introduction of TACT. The National Malaria Control Programme (NMCP) is considering adopting TACT as a second-line therapy in their national treatment guidelines. This will make it easier for them to rapidly switch to TACT in case artesunate-mefloquine (ASMQ) would fail again followed by AS-PYR failures. Other countries in the GMS should consider similar pro-active strategies instead of relying on reactive approaches towards drug resistance [4, 18]. Furthermore, respondents suggested that sufficient stockpiles of effective therapies, even beyond malaria elimination, is required to prevent resurgence of the disease. Stocking challenges have indeed been widely acknowledged in literature on anti-malarial drug transitions [22, 23], and have often been associated with expiry dates [21, 24], or low expected demand [25]. National malaria control programmes in the GMS should take a guiding role in ensuring adequate distribution and stocking of malaria therapies given their wealth of experience during previous drug transitions [15]. Beyond national level policy guidance, interview respondents considered international organizations, such as the WHO, essential to guide the potential introduction of TACT through recommendations, guidelines and resource dedication. This implies the necessity of a pro-active role for these policy institutes, similar to the transition to ACT in the early 2000s [15].

The second strategic theme that emerged from the study was related to the collection of data and evidence on safety and efficacy. Before TACT can be deployed, evidence from clinical trials will be required to obtain market authorization in Cambodia, Vietnam and Lao PDR. Regulatory procedures have however repeatedly been suggested as delaying factors in updating malaria treatment routines [15, 26]. They are often considered inflexible, while dealing with drug resistance requires pragmatic approaches [27]. Established regulatory procedures entail large-scale clinical studies and approval by a stringent regulatory authority – such as WHO pre-qualification – before country-level authorization procedures can commence. Interview respondents emphasized that strategies for early dossier submission should be explored to shorten regulatory timelines [27]. Similar procedures were suggested for reducing implementation timelines for TACT in Nigeria and Burkina Faso [28]. One pragmatic strategy was suggested by interview respondents in Vietnam. They referred to the current off-label prescription of AS-PYR prior to its country-level registration because a new therapy was urgently needed after DHA-PPQ failures were observed.

The third strategic theme that emerged was related to logistical and operational considerations. Although malaria incidence in the GMS has significantly been reduced in the last decades, references were made to persisting general malaria management and control challenges. Malaria in the GMS is mostly prevalent amongst hard to reach populations in remote border areas, such as forest workers and labour migrants [29, 30]. New interventions will need to reach these remote populations and they will need to be adequately adopted in order to contribute to malaria control and elimination strategies. Community-based health initiatives such as Village Malaria Worker (VMW) programmes have become a key intervention in malaria control strategies in the GMS [31]. They are now institutionalized in public health services and are considered essential to the battle against malaria. VMWs should be actively involved in the potential introduction of TACT which will require policy attention and resource dedication [4, 32]. Another prominent challenge that was identified by interview respondents are the limited incentives for manufacturers to engage in TACT. It was emphasized that the low case incidence of falciparum malaria in the GMS would translate to low sales volumes and that this could deter manufacturers to engage in triple artemisinin-based combination production. However, history has shown that this market dilemma can successfully be addressed, for example through corporate social responsibility programs, public–private partnerships, or external funding for research and development efforts [15, 26, 33–35]. Another solution that was mentioned during the interviews was a regional pooled procurement system for the GMS. Such a pooled procurement system would enable to reach scale advantages. The GFATM could be a credible partner to rollout such a scheme, given their wealth of experience in reforming global health procurement systems [36]. Further distribution challenges in Vietnam and Cambodia were considered limited because these countries now rely on public sector channels which are relatively compliant to national guidelines [15]. More challenges were expected for Lao PDR where private sector distribution channels play a significant role in malaria management efforts. Lessons from earlier private sector engagement programs, such as the Public–Private Mix (PPM) [37] and the Affordable Medicines Facility malaria (AMFm) initiative [36, 38, 39] should therefore be evaluated and translated to the potential introduction of TACT.

The fourth and final strategic theme was downstream engagement for TACTs deployment. A recurrent question that emerged is how to engage stakeholders in TACTs while falciparum malaria incidence is receding in the GMS. Changing first-line treatment practices indeed requires substantial investments and collective actions by multiple stakeholder groups such as decision makers, regulators and prescribers [28]. Antimalarial drug transitions are lengthy and this justifies considerations around the timing of introducing new therapies. Previous studies have emphasized that downstream engagement strategies are important to the successful introduction of new therapies [21, 40]. Therefore, adequate training materials should be developed for TACTs. Such training modules should be clear and discuss risks of potential side-effects [21, 41]. Moreover, respondents stressed that training programs should include education messages to address potential misconceptions about the new therapy. For the introduction of TACTs, communication strategies were also deemed necessary which should highlight advantages in terms of clinical benefits, TACTs’ potential to protect antimalarial drugs, and indirect benefits such as reduced frequency of policy changes [14].

Four limitations should be considered when interpreting the study results. An important disclaimer is that this study does not seek to determine whether TACTs should be introduced in the GMS; we present strategic considerations for the market introduction of TACTs. Second, the generalizability of the findings is limited. Although some of the findings of this study are also relevant to other countries in the GMS, countries are heterogeneous and characterized by their own healthcare systems and epidemiological factors. We propose that similar studies should be conducted in other countries that are confronted by drug-resistant malaria. Third, although we selected respondents in close collaboration with local research institutes, it is possible that important stakeholders and perspectives were not included. Fourth, interviews were conducted in local languages and translated into English, hence translation bias could have occurred.

This paper presented a qualitative study regarding strategies to identify implementation challenges for deploying triple artemisinin-based combination therapy (TACT) and to discuss strategies to overcome these challenges in three countries in the Greater Mekong Subregion (GMS). It explored considerations for deploying TACT around four strategic themes: policy support, data and evidence, logistics and operation, and downstream engagement. The findings could benefit researchers and decision makers in strategizing effectively towards potential future deployment of TACT in the GMS.

## Data Availability

The datasets used and/or analysed during the current study are available from the corresponding author on reasonable request.

## Abbreviations

ACT: Artemisinin-based combination therapy
AL: Artemether-lumefantrine
AL + AQ: Artemether-lumefantrine plus amodiaquine
ASEAN: Association of Southeast Asian Nations
ASMQ: Artesunate-mefloquine
AS-PYR: Artesunate-pyronaridine
CMPE: Center of Malariology, Parasitology and Entomology, Lao PDR
CNM: National Center for Parasitology, Entomology and Malaria Control, Cambodia
DAV: Drug Administration of Vietnam
DDF: Department of Drugs and Food, Cambodia
DHA-PPQ: Dihydroartemisinin-piperaquine
FDD: Food and Drugs Department, Lao PDR
FDC: Fixed dose combination
GFATM: Global Fund to fight Aids, Tuberculosis and Malaria
GMS: Greater Mekong Subregion
NMP: National Malaria Program, Vietnam
PPM: Public–Private Mix program, Lao PDR
TACT: Triple artemisinin-based combination therapy
VMW: Village malaria workers
WHO: World Health Organization
